# A Review of the Impact of Starch on the Quality of Wheat-Based Noodles and Pasta: From the View of Starch Structural and Functional Properties and Interaction with Gluten

**DOI:** 10.3390/foods13101507

**Published:** 2024-05-13

**Authors:** Jinrong Wang, Yonghui Li, Xiaona Guo, Kexue Zhu, Zijian Wu

**Affiliations:** 1 College of Biotechnology and Food Science, Tianjin University of Commerce, Tianjin 300134, China; 2 Department of Grain Science and Industry, Kansas State University, Manhattan, KS 66506, USA; yonghui@ksu.edu; 3 State Key Laboratory of Food Science and Technology, Jiangnan University, Wuxi 214122, China; xiaonaguo@jiangnan.edu.cn (X.G.); kxzhu@jiangnan.edu.cn (K.Z.); 4 School of Food Science and Technology, Jiangnan University, Wuxi 214122, China; 5 Key Laboratory of Low Carbon Cold Chain for Agricultural Products, Ministry of Agriculture and Rural Affairs, Tianjin 300134, China

**Keywords:** noodles, quality, starch, interaction between starch and gluten

## Abstract

Starch, as a primary component of wheat, plays a crucial role in determining the quality of noodles and pasta. A deep understanding of the impact of starch on the quality of noodles and pasta is fundamentally important for the industrial progression of these products. The starch structure exerts an influence on the quality of noodles and pasta by affecting its functional attributes and the interaction of starch–gluten proteins. The effects of starch structure (amylopectin structure, amylose content, granules size, damaged starch content) on the quality of noodles and pasta is discussed. The relationship between the functional properties of starch, particularly its swelling power and pasting properties, and the texture of noodles and pasta is discussed. It is important to note that the functional properties of starch can be modified during the processing of noodles and pasta, potentially impacting the quality of the end product, However, this aspect is often overlooked. Additionally, the interaction between starch and gluten is addressed in relation to its impact on the quality of noodles and pasta. Finally, the application of exogenous starch in improving the quality of noodles and pasta is highlighted.

## 1. Introduction

Noodles have been a staple food in Asian cuisine for millennia. They are usually prepared from unfermented dough by stretching, extrusion, or rolling processes before being cut into various shapes [1]. Pasta is a type of noodle of Italian origin, made from an unleavened dough of durum wheat flour [2]. The profound cultural interplay between nations has cultivated a rich array of noodles and pasta that are adored globally. Presently, the quest for superior noodle varieties is spurred by many factors, ranging from gustatory delight to health imperatives, precipitating a surge in research endeavors aimed at enhancing the refinement and nutritional value of noodles and pasta.

The quality of noodles and pasta attributes mainly include color, cooking properties (cooking time, cooking loss, water absorption), texture characteristics (firmness, elasticity, chewiness, and stickiness) and sensory quality [3]. The color of noodles and pasta is usually detected by a colorimeter. Noodles of different types present different surface colors. The high-quality white salted noodles, such as Udon, show a bright, creamy appearance [4]. In addition, a yellow surface is acquired for alkaline noodles, like Japanese ramen and Cantonese noodles [1]. Pasta of a high quality usually presents a bright yellow appearance [5]. The cooking property is usually determined according to standard methods, such as the AACC Method 66-50.01 [6]. Consumers mostly prefer noodles with a shorter cooking time and a lower cooking loss.

A texture analyzer is extensively employed to ascertain the textural characteristics of noodles and pasta [7]. Consumers’ expectations regarding the textural attributes of noodles are considerably intricate. Individuals hailing from diverse regions exhibit varying preferences when it comes to texture requisites. Chinese people prefer noodles that are characterized by a firm, resilient, and smooth texture, while noodles with a soft texture enjoy greater favor in Japan, such as Japanese white salted noodles [7]. It can be inferred that noodles of varying varieties differ in color, texture, and sensory attributes. Moreover, the expectations regarding noodles and pasta quality among consumers in diverse locales exhibit distinct variations. Consequently, comprehending the effect mechanism of noodles and pasta quality is crucial for crafting noodles and pasta of heightened desirability.

Gluten and starch stand as the primary constituents of noodles. Gluten protein forms a gluten network during the dough-making process, and this network serves as the skeleton of the dough and the final product. Unquestionably, the gluten protein bestows a distinctive influence upon noodles and pasta quality. Starch, embedded in the network, does not simply serve as filler. It also plays a crucial role in determining noodles and pasta quality [8]. Li et al. [7] reviewed the impact of starch on noodle quality, highlighting that the variations in functional properties (such as swelling, pasting, and gelatinization), caused by starch structure, can account for differences in noodles texture. Noodles and pasta constitute a composite entirely comprising starch and gluten protein. The structural and functional properties of starch not only directly influence the quality of noodles and pasta, but also exert influence through modifications in the protein network framework [9]. This elucidates the divergent research discoveries concerning the impact of starch on noodles and pasta caliber across distinct research groups. Notwithstanding, the significance of the starch–gluten interaction is frequently undervalued.

To holistically grasp the impact of starch on noodles and pasta quality, the correlation between starch properties and noodle/pasta quality (color, water absorption, optimum cooking time, and the cooking loss of raw noodles, as well as the texture and sensory quality of cooked noodles) is delineated. Furthermore, the following mechanism is reviewed on the aspects of the functional characteristics of starch and the interplay between starch and gluten, with the goal of providing a thorough point of reference for forthcoming research in this domain.

## 2. Difference between Noodles and Pasta on Raw Material, Processing, and Quality

Diverse regional cultures, dietary traditions, and technological methodologies contribute to unique variations in raw materials, processing techniques, and quality benchmarks for noodles and pasta. Noodles predominantly rely on common wheat flour as their principal component, whereas pasta prominently features semolina derived from durum wheat. Specifically, the particle size of semolina used in pasta production typically ranges between 150–550 μm, contrasting with the wheat flour utilized in noodle-making, which boasts D50 values below 100 μm [10,11]. The distinguishing factor between durum and common wheat rests largely in their protein compositions. Durum typically boasts an elevated protein content and reduced starch content. The protein in durum also has elevated ratios of HMW/LMW (High Molecular Weight/Low Molecular Weight), contributing enhanced strength to pasta dough [12]. Compared to common wheat, the starch in durum usually has a lower lipid content, leading to an increased gelatinization temperature [13]. To enhance the nutritional value and quality of noodles and pasta, various ingredients such as grains (e.g., oat, barley, buckwheat), vegetables (such as spinach, tomato), fruits, tea, eggs, exogenous proteins (like soy isolate protein), and native additives (such as starch, edible gums, enzymes, organic acids, natural polyols) are incorporated during production [2,14,15]. Moreover, certain salts (such as NaCl, Na_2_CO_3_) are utilized to improve noodle quality, a practice comparatively uncommon in pasta production [2].

Distinct processing methodologies differentiate noodles and pasta. Noodles commonly undergo sheeting, cutting, or stretching processes, while pasta is typically produced through extrusion or sheeting. Notably, extrusion is favored in industrial pasta production for its heightened efficiency and versatility [16]. Surface color, cooking attributes, and texture also distinguish pasta from noodles. Pasta often showcases a yellow hue, owing to carotenoids present in durum wheat, whereas noodles exhibit varying surface colors. Alkaline noodles, like lamian, exhibit a yellow appearance, contrasting with the white color of salt noodles. Differences further manifest in cooking characteristics and sensory qualities: pasta typically requires a longer cooking time than noodles and delivers a firmer texture [17].

## 3. General Structure of Starch

Starch, stored in plant roots, seeds, stems or even flowers, consists of amylose and amylopectin. Normally, amylose constitutes 20–30% of the starch weight, whereas amylopectin makes up 70–80%. Amylose is a linear molecule, composed of 500 to 6000 glucose residues. Most of these glucose molecules are connected by α-(1-4) glycosidic bonds [18]. However, a few short branches have been found in amylose molecules. These short branches are connected to a backbone chain by α-(1-6) glycosidic bonds, manifesting at a rare frequency [18]. Most amylose molecules are arranged in a disordered conformation. A few amyloses formed complexes with lipids and present V-type X-ray diffraction spectra. Several arguments have been put forward regarding the accurate location of amylose within starch granules. Reports suggest that a significant portion of amylose molecules are presumed to reside within the amorphous or less-crystalline regions of starch granules [19], while some reports indicated that amylose molecules were mainly found in the granule cores [20].

**Figure 1:**
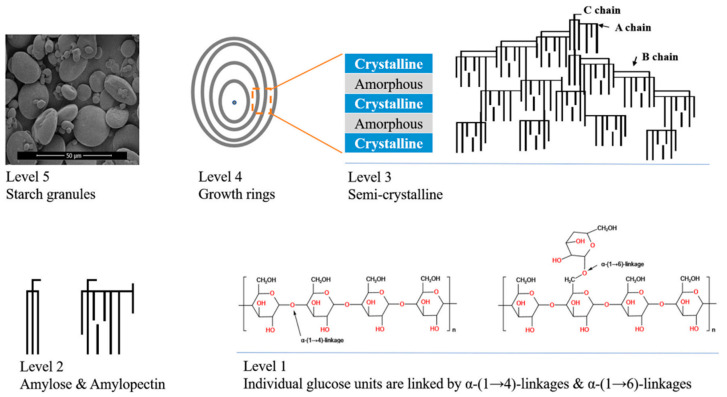
Multilevel structure of starch. Reproduced with permission from Huang, Wang, Fan, Ma, (2022) [25].

Amylopectin is also composed of glucose residues. However, in contrast to amylose, amylopectin is a highly branched molecule with a higher molecular weight, ranging from 10^7^ to 10^9^ g/mol [18]. The glucose units in amylopectin are connected by 95% α-(1, 4) linkage and 5% α-(1, 6) linkage, and the α-(1, 6) linkage is the branch point. Several models for amylopectin have been proposed in previous studies, of which the “cluster” model is the most widely accepted [18,21]. The branches of amylopectin are classified into A-, B-, and C-type chains (Figure 1), with chain lengths ranging from 6 to 100 glucose residues. Each amylopectin molecule has only one C-type chain, which is located at the center of the amylopectin molecule and possesses the sole reducing end of amylopectin. A-type chains are at the outermost of amylopectin. These chains are linked with B-type chains through α-(1, 6) glycosidic. The B-chains are further classified into B_1_, B_2_, B_3_, B_4_ chain subtypes [22].

A-chain and B_1_-chains together form the double helixes. Most of the double helixes orderly arrange into crystalline lamellae, and the other part of amylopectin is in the amorphous lamellae. The crystalline and amorphous lamellar together form a repetitive lamellar structure with a thickness of about 9 to 10 nm, as determined by Small Angle X-ray Scattering. It is believed that the semi-crystalline growth ring is formed by the periodic lamellar structure of 9–10 nm. The starch granule is formed by the semi-crystalline growth ring and the amorphous growth ring. Relatively few studies have described the amorphous growth ring [23]. Starch granules, formed by semi-crystalline and amorphous growth rings, display a semi-crystalline structure, determined by X-ray diffractometer. When observed by SEM (scanning electron microscopy), starch granules usually present spherical, dish-shaped, oval, or irregular shapes. Wheat starch is composed of dish-shaped A-granules and spherical B-granules, and obvious pores and grooves can generally be observed on the A-granule [24].

## 4. Effect of Starch on the Quality of Noodles and Pasta

### 4.1. Starch Structural Properties

As described above, starch exhibits multilevel structures. Concurrently, starches derived from various wheat species diverge in their structural properties, including the amylopectin fine structure, amylose content, granule size distribution, and crystalline structure [24]. In addition, different wheat milling technology and the hardness of wheat would also result in a changed damaged starch level [26]. As expected, the starch structure properties have a significant effect on the quality of noodles and pasta. The effects of amylopectin structure, amylose content, granule size distribution, and damaged starch content on the quality of noodles and pasta have been widely investigated. However, the influence of long-range order (crystalline structure) and the short-range order of starch are rarely documented [7]. This is because the long-range and short-range order of native starch is directly affected by the structure of amylopectin, the ratio of amylose to amylopectin, etc. [27], and these long-range and short-range orders of starch would be severely destroyed during cooking process [7]. As a result, only the effects of the amylopectin structure, amylose content, granule size distribution, and damaged starch content on quality of noodles and pasta are reviewed this paper.

#### 4.1.1. Amylopectin Structure

Amylopectin, a pivotal constituent of starch, constitutes 70–80% of normal starch. The molecular characteristics of amylopectin, such as molar mass, molecular dimensions or size (radius of gyration), molecular density, branching degree, and the distribution of short-chains, have obvious effects on starch physicochemical properties [28,29] these effects may further influence the quality of noodles and pasta. Enhancing the amylopectin content appropriately can elevate the cooking quality, sensory attributes, and textural properties, as detailed in Section 4.1.2 of this paper.

The effect of amylopectin structure on the cooking properties of noodles and pasta is seldomly reported, while the effect of amylopectin structure on texture properties of noodles obtains more and more interesting reports in recent years. The amylopectin fine structure is found to affect the texture properties of cooked noodles [30]. The amount of the long-branched amylopectin chain has a positive effect in enhancing the hardness of noodles [31]. The amylopectin also has a direct effect on the smoothness of noodles. Exactly, the amylopectin leached during the noodle cooking process would attach to the noodle surface, which plays key role in the smoothness of noodles. The leached amylopectin with longer chain length distribution could form a dense gel network with amylose, and its branch chains could also combine with more water molecules through hydrogen bonding to produce gel. As a result, amylopectin, with a longer chain length distribution, has a positive effect on the smoothness of noodles [32]. Research from Barbiroli et al. (2013) [33] suggests that alterations in the amylopectin structure during pasta processing (primarily extrusion and drying) significantly influences the quality of pasta. However, the exact nature of these changes remains unclear. The effect of the amylopectin structure on noodles and pasta quality may be related to its impact on starch thermal properties, like swelling, solubility, and pasting. The effect of amylopectin structure on other qualities of noodles/pasta, such as cohesiveness, springiness, gumminess, is seldom reported.

#### 4.1.2. Amylose/Amylopectin Ratio

The amylose/amylopectin ratio exerts a substantial influence on starch structure (long- and short-range order, granules size distribution) and functional properties (e.g., gelatinization, pasting, and swelling property) [34,35], thus, the quality of noodles and pasta (Table 1).

As summarized in Table 1, the amylose/amylopectin ratio distinctly impacts the cooking properties of noodles. The elevated amylose/amylopectin ratio tends to prolong the cooking duration and reduce the water uptake of noodles/pasta. The extended cooking time predominantly stems from heightened pasting or gelatinization as the amylose content increases [40], while the opposite result is also observed in pasta cooking time [37]. On the other hand, the increased amylose/amylopectin ratio has a positive effect on enhancing the resistance to overcooking, which may relate to the lower swelling power of starch with a higher amylose content [37,38]. The diminished water absorption of noodles results from the constrained swelling of starch, caused by the heightened amylose content [44]. The amylose/amylopectin ratio positively correlated with the cooking loss of noodles [38].

As reviewed in Table 1, the elevated amylose/amylopectin ratio exhibits a positive correlation with the firmness of cooked noodles and pasta. This enhanced firmness arises from the densely packed starch granules with a reduced swelling capacity, characteristic of a higher amylose content [31,36]. The cohesiveness of noodles exhibits a pronounced upward trend as the amylose content escalated from 21% to 30%, while the springiness and resilience remained largely unaffected [44]. Nevertheless, alternative studies suggest a negative correlation between the cohesiveness, springiness, and the resilience of cooked noodles and the amylose content of starch [36,40]. It can be confirmed that the amylose/amylopectin ratio content exhibits a positive correlation with the firmness of noodles and pasta. However, the link between the amylose/amylopectin ratio and other textural aspects of noodles and pasta (springiness, resilience, smoothness, cohesiveness, etc.) remains ambiguous. This ambiguity may arise from the possibility that the influence of the amylose/amylopectin ratio on springiness, resilience, smoothness, cohesiveness could be diminished or even obscured by other contributing factors. An optimal balance between amylose and amylopectin is imperative for enhancing the sensory quality of noodles and pasta. Zi et al. (2019) [8] propose that noodles prepared from wheat flour with 18.5–21.5% amylose show good sensory qualities. A greater amylose/amylopectin ratio may be required for pasta of a superior sensory perception, as consumers typically lean towards pasta characterized by heightened firmness. A recent investigation revealed that the structure of amylose, including factors like chain length and branching, significantly impacts the quality of noodles [31], while the influence of amylose structure on food quality is frequently overlooked.

#### 4.1.3. Starch Granule Size Distribution

Starch granules exhibit varied morphologies, ranging in size from 1 to 100 µm. Both common and durum wheat starch consists of A-type granules (large) and B-type granules (small) [24,45]. These distinct granules undergo biosynthesis at varying intervals post-anthesis, with A-granules emerging initially, followed by the later appearance of B-granules [25]. Previous investigations suggest that wheat starches display fluctuations in the ratios of A-granules to B-granules across various genotypes [46]. Variances are identified within the structural aspects (granule structure, crystal structure, and amylopectin structure) [47] and functional attributes (gelatinization and retrogradation) [48] between the A-type and B-type starch granules. Hence, the ratio of A-granules to B-granules plays a crucial role in determining the quality of noodles and pasta, which has been investigated widely [47,49,50].

Within the dough of pasta and noodles, starch is encased within the three-dimensional framework created by gluten though covalent reactions and some non-covalent interactions (hydrogen bonding and hydrophobic interactions) [51,52]. Gluten serves as the skeletal structure, with gliadin functioning as the plasticizer of gluten [53]. Starch granule structure affects the gluten network structure in dough, consequently shaping the rheological characteristics of noodles and ultimately defining the final quality [54]. Typically, small B granules are arranged densely in the dough, a configuration advantageous for constructing the gluten matrix. As a result, an increased concentration of B granules leads to enhanced dough firmness but reduced elasticity [49,55,56]. The favorable impact on the gluten matrix also contributes to the improved cooking quality of noodles and pasta. B-granules play a role in reducing the cooking loss of noodles and pasta [47,49,55]. Nevertheless, the proportion of B-granules in noodles with the least cooking loss has exhibited variability across studies. Additionally, the compact arrangement of small granules enhances the lightness of white salted noodles [55].

The firmness, chewiness, and viscoelasticity of white noodles demonstrate an increase alongside the escalation in small granule content [49,55]. Similar trends are observed in pasta [47,57]. It is worth highlighting that white noodles achieve the optimal texture and sensory ratings when the B-granule proportion approximates 40% [49,55,58]. Moreover, durum wheat with a B-granule content ranging from 32% to 44% is notably more appropriate for pasta production [57]. The tight arrangement of small starch granules (B-type starch granules) and the consequent robust gluten network architecture serves as the underpinnings for the superior quality of noodles and pasta. Furthermore, the disparity in thermal characteristics (swelling capacity, pasting attributes) of starch due to diverse particle dimensions influenced by structural factors (including amylose content, amylose form, amylopectin configuration, among others) also contributes to the diversity in noodles and pasta quality.

#### 4.1.4. Damaged Starch (DS) Content

Damaged starch typically emerges from mechanical strains experienced during wheat milling or comparable processing phases. The quantity of damaged starch present in flour is influenced by milling conditions, such as milling time, milling power, moisture content, and also the hardness of the wheat kernel [59]. Moreover, specialized processing methods, like low-temperature drying and pasta extrusion can also induce the creation of damaged starch [60,61]. Damaged starch granules are more susceptible to enzymatic hydrolysis than their natural counterparts, which can be evaluated by an enzymatic colorimetric micro method [62]. It can also be detected using the iodometric method, and by assays using a near-infrared reflectance technique [59]. Comparable to the impact of starch granule size, the presence of damaged starch in the raw material initially influences dough rheology [63], subsequently impacting the quality of noodles (Table 2).

Typically, a greater presence of damaged starch corresponds to a higher water absorption capacity, hindering gluten hydration and ultimately diminishing the quality of the end product. In the realm of pasta manufacturing, elevated levels of damaged starch can adversely impact gluten hydration processing [16]. Moreover, heightened damaged starch levels catalyze the production of furosine [64]. Consequently, in pasta production, medium-to-large semolina particles with reduced damaged starch content are preferred [16]. The impact of damaged starch on pasta quality is rarely documented. Interestingly, the damaged starch content exhibits clear upward trajectories during pasta processing, notably during extrusion and low-temperature drying [61,65]. Nevertheless, the result of this heightened damaged starch content on pasta quality remains inadequately understood [61].

The damaged starch content is an important factor of flour, which is of significant effect on quality of food. During the early stages, researchers primarily utilized varied milling conditions to prepare wheat flour with differing levels of damaged starch content, employing these to investigate the impact of damaged starch on the quality of noodles or pasta (Table 2). The presence of damaged starch has the potential to enhance the brightness of noodles [66]. This heightened luminosity can be attributed to the increased reflectivity of flour containing elevated levels of damaged starch, owing to its typically expanded surface area. Pasta prepared from semolina with low damaged starch level content may result in an amber-yellow color and low heat damage [16]. The content of damaged starch demonstrates a detrimental effect on the water absorption of noodles and a beneficial impact on cooking loss values. This correlation may stem from the damaged starch granules’ inability to maintain structural integrity during the cooking process [67]. However, these influences of DS, brought by the milling process, are likely overshadowed by various other factors, including the particle size of the flour, ash content, flour grade, the levels of flavonoids, and the introduction of alkaline substances [66,67,68].

**Table 2:** Effect of the damaged starch content on the quality of wheat flour-based noodles.

Type of Noodles	Factors Leading to Variations in Damaged Starch Content	DS Content (%)	Effect of Damaged Starch on Quality of Noodles	Reference
Chinese fresh white noodle	Reconstituted flour	5.5–10.4	Increased noodle hardness and springiness	[44]
Noodles	Flour milled using the ultrafine pulveriser with power varied from 45 to 130 Hz	6.54–12.06	Non-linear effect on noodle color and texture	[66]
Yellow alkaline noodle	Flour milled using either fluted or frosted reduction rolls at various roll differentials and roll gaps	-	Positive effect on texture of noodle with kansui Negative effect on texture of noodle with NaOH	[67]
Yellow alkaline noodle	Flour milled using either fluted or frosted reduction rolls at various roll differentials and roll gaps	-	Positive effect on noodle elasticity	[68]

-, signifies that the data were not reported. DS: Damaged starch.

An optimal level of damaged starch (DS) has the capacity to augment the firmness and elasticity of noodles. This outcome is linked to the reinforcement of dough resilience resulting from an optimal DS level, as DS facilitates enhanced water absorption within the dough. Conversely, an excessive DS level results in the deterioration of textural properties, a consequence of the disruption caused by fine grinding [68]. Masato et al. [61] suggest that the heightened presence of damaged starch during pasta processing may also exert a substantial influence on pasta texture, owing to its impact on starch swelling and gel properties. The impact of damaged starch level on noodle quality has also been investigated using reconstituted flour. The firmness of white noodles prepared from reconstituted flour shows an uptrend as the damaged starch levels elevate from 5.5% to 10.4%, with an opposing trend emerging as damaged starch levels continue to escalate. The springiness of noodles made from reconstituted flour is discovered to heighten with augmented damaged starch content, concurrent with a decline in cohesiveness [44]. A marginal elevation in damaged starch content resulted in a minor uptick in the sensory rating of noodles, whereas a marked decrease in noodles’ sensory rating becomes apparent when the damaged starch exceeds 8% [66].

During the 1980s–2010s, the quality of wheat flour, like damaged starch, particle size, and farinograph properties, garnered significant attention. Consequently, the investigation into the impact of damaged starch on the quality of noodles is widely pursued in early times. Wang et al. [59] suggest that damaged starch should be controlled at a moderate level for food processing. With the evolution of noodles and pasta processing methods and deeper investigations, it has been revealed that damaged starch emerges during various stages of noodles and pasta production, including dough mixing and resting [69], storage [70], and pasta extrusion [71]. Despite this, a conclusive understanding of the influence of DS generated during processing on the quality of noodles and pasta remains unclear. Greater attention should be given to the formation of damaged starch during noodles and pasta processing.

### 4.2. Starch Functional Properties

The variation in starch structural properties would first lead to changes in the functional properties, and subsequently affecting the quality of noodles and pasta. Moreover, a clear correlation has been noted between starch functional properties, especially pasting and swelling power, and the quality of noodles and pasta [7].

#### 4.2.1. Pasting Property

The pasting characteristics of starch are evidently intertwined with the quality of noodles. The parameters of starch pasting properties, like pasting viscosity, breakdown, and final viscosity, exhibit a positive correlation with the softness and elasticity of white salted noodles [31,72]. The starch pasting temperature demonstrates a negative correlation with the smoothness and sensory of white salted noodles [8]. Furthermore, it has been observed that starch exhibiting an appropriate peak viscosity is advantageous for enhancing the texture and sensory quality of white salted noodles [8,73]. The influence of starch pasting properties on alkaline-noodles quality may vary, as the pasting properties of starch are notably impacted by alkaline [74,75].

The investigations on the correlation between pasting properties and noodles/pasta quality typically involve the use of wheat flour with varying amylose content. Nevertheless, it is worth noting that the pasting properties of starch can be altered by certain additives, such as phosphate salts and alkaline substances [74,76,77] as well as through noodles and pasta processing techniques, like high-temperature treatment [78], fermentation [79], prolonged storage [70], and extrusion, etc. [65]. Greater consideration should be directed towards the repercussions of modified starch pasting characteristics arising from additives and processing methodologies on the quality of noodles and pasta [3,74].

#### 4.2.2. Swelling Behavior

The swelling behavior of starch can be treated as the water absorption capacity of starch at a specified temperature. It is primarily influenced by the amylose content, amylopectin structure, granule size distribution, lipid content, amylose-lipid complex, damaged starch, and the ordered structure of starch [24,80]. Moreover, the subtle effects of starch swelling behavior on noodles and pasta quality have been examined in previous studies.

The softness, elasticity and smoothness of white noodles and yellow alkaline noodles are positively correlated with the starch swelling power [31,63,81]. Moreover, the firmness of pasta diminishes with the escalated starch swelling power [82,83]. The underlying mechanism detailing the impact of starch swelling on the quality of noodles and pasta may be elucidated by the heightened absorption of starch water as the swelling power intensifies. This process contributes to rendering noodles and pasta softer, smoother, and more elastic. Moreover, the high swelled starch will occupy additional space and impede the cross-linking of gluten, thereby leading to a softer consistency in noodles and pasta [52,84].

## 5. Interaction between Starch and Protein

Starch interacts with the gluten complex through both physical and chemical bonding [85,86,87], a phenomenon observable during the dough mixing and cooking processes of noodles and pasta preparation. Additionally, the interaction between starch and gluten noticeably impacts the quality of noodles and pasta. These interactions are also influenced by alterations in the starch structure.

**Figure 2:**
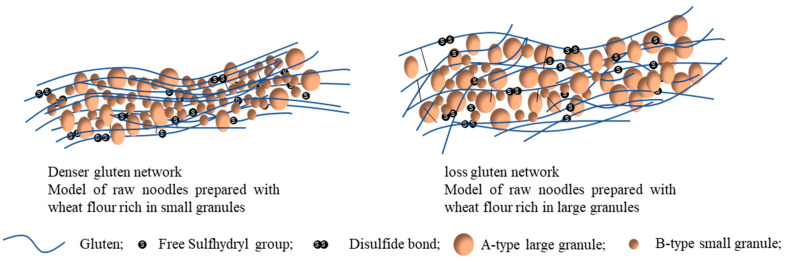
Model structure of the noodles prepared using wheat flour with different starch granule size distribution.

These interactions of starch/protein at the stage of dough mixing are impacted by many factors, such as amylose/amylopectin ratio, starch granular size, damaged starch and gluten composition. The effect of the amylose/amylopectin ratio on the interaction of starch/gluten is linked to its effect on water absorption. A higher amylopectin content can augment the water absorption capacity and reinforce the competitive nature of starch against gluten during dough mixing [88,89]. Li et al. [42] further propose that non-covalent bonds are established between starch granules and gluten, exerting an influence on the texture and digestibility of noodles. Variations in the distribution of starch granule sizes lead to a range of starch/protein interaction areas and strengths [8,86,90]. Increasing the presence of small starch granules augments the interaction area between starch and protein [30]. Initially, this effect boosts mixing properties, water absorption, dough strength, and the firmness of noodles and pasta [56]. Recent research by Yan and Lu, (2021) [49] suggests that starch granules play a role in shaping the gluten network through physical or chemical interactions. Moreover, a higher percentage of small granules can significantly densify the gluten network, resulting in an upward trend in noodles firmness [91]. Shang et al. [91] suggest that starch interacts with gluten via non-covalent bonds, whereby an increase in B-type starch granules intensifies hydrogen interactions while decreasing hydrophobic interactions. Consequently, this results in an elevated β-structure and ultimately enhances noodle quality. The model depicting noodles prepared using wheat flour with varying starch granule sizes is illustrated in Figure 2. Unlike B-type starch, increased damaged starch can disrupt the bond within gluten due to leached starch chains from the damaged starch [92]. This may due to the free hydroxyl group, which occurred during mechanical activation, and can interact with the sulphydryl groups of cysteine residues in the polypeptides of gluten protein, leading to decreased disulfide bonds. It is also noted that starch interacts with gluten in the dough through hydrophobic forces, which can be mitigated by a higher gliadin content at an optimal mixing phase [93].

**Figure 3:**
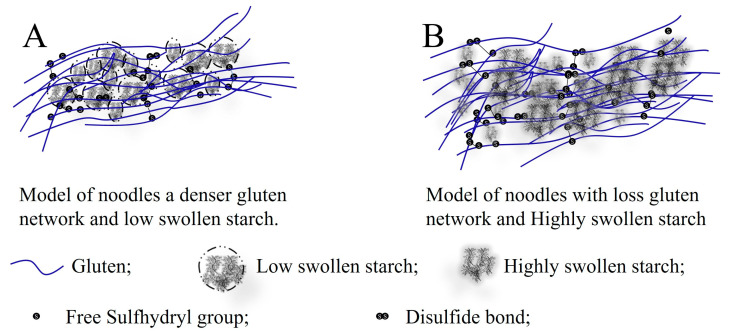
(**A**,**B**) Model structure of cooked noodles containing starch of different swell degree.

An intricate interplay unfolds during the cooking process. Initially, starch competes for water alongside gluten throughout the cooking phase. This rivalry impedes the gelatinization of starch and the aggregation of protein. Subsequently, the expansion of starch gives rise to a “steric hindrance” on the amalgamation of gluten, a phenomenon affected by the structures of both starch and protein. For instance, an elevated amylose content in starch constrains starch expansion, thereby reducing the space it occupies during cooking [42]. Hence, there is a greater expanse for protein to establish a robust framework [52], as depicted in Figure 3A. This phenomenon leads to a heightened rigidity, extended cooking duration, and diminished cooking losses and water absorption. The enhanced rigidity is, to some extent, a consequence of the heightened interaction between amylose and protein [94]. Conversely, starch with a lower amylose content or a greater swelling capability occupies a larger volume during cooking, hindering the formation of the protein framework [84]. Consequently, this results in reduced rigidity, shorter cooking times, increased cooking losses, water absorption, and elasticity. Additionally, the characteristics of gluten also impact the competition for water and space with starch. The study conducted by Jekle et al. [95] suggests that gluten serves primarily as a hindrance to starch swelling, followed by its involvement in battling for moisture. Gluten with a swifter aggregation capacity or elevated content restrains the absorption of water and the swelling of starch, resulting in enhanced textural qualities and an increased resistance to starch digestion [95,96]. Following starch gelatinization, starch chains diffuse outward and intertwine with gluten through non-covalent and covalent bonds, which is impacted by starch and gluten properties [97]. Hydrogen bonds may develop between the second or third free hydroxyl group of glucose in the starch strand and glutamine in gluten [98]. According to Kuang et al. [99] starch chains interact with gluten through hydrogen bonds and hydrophobic interactions, disrupting gluten aggregation and leading to decreased gluten digestibility. Throughout the heating phase, diffused starch chains also interact with gliadin or globulin through isomeric glycosidic bonds [100]. The formation of isomeric glycosidic bonds is influenced by the chain structure, with shorter chains exhibiting a greater propensity to form such bonds with proteins.

At present, research on the impact of starch–protein interaction primarily focuses on spatial and water competition. Zhang et al. [101] suggests that achieving the appropriate equilibrium between starch and gluten networks stands as the pivotal determinant for noodle quality. It is essential to meticulously ascertain the optimal equilibrium for various types of noodles and pasta while unraveling how to characterize the interplay between starch and protein. Beyond space and water competition, starch also forms chemical bonds with protein, influencing gluten conformation, aggregation, and consequently, the quality of noodles and pasta. Nonetheless, the precise influence of these chemical interactions on the quality of noodles and pasta remains unknown, with only limited research currently available.

## 6. Application of Starch in Improving Noodles and Pasta Quality

Many methods have been employed to improve the quality of noodles/pasta by manipulating the properties of starch, such as breeding, adding exogenous starch, and others. Among these, breeding stands out as the most intricate and time-intensive method. In contrast, the addition of exogenous starch represents a comparatively straightforward technique. Additionally, alternative method, such as process modifications and the introduction of food additives, are also commonly utilized.

### 6.1. Breeding

Quality trait is an important target for breeding, and a lot of research about breeding wheat variety suitable for noodles and pasta has been conducted [4,102,103]. To date, a lot of investigations have delved into the art of breeding specifically for Udon or the traditional Japanese white salty noodles. These studies focus on modulating amylose content, viscosity, and starch swelling by modulating the expression of granule-bound starch synthase I (GBSSI) proteins accountable for amylose production, as well as the trio of starch synthase II (SSIIa) enzymes crucial in amylopectin generation [37,38,41]. In general, the Wx-B1 allele exerts a greater effect on amylose content within starch. Currently, there exist instances where noodles and pasta quality has been enhanced through selective breeding. For example, research conducted by Zi et al. [104] shows that the absence of Wx-B1 results in diminished amylose levels, subsequently elevating the overall quality of the noodles. Furthermore, the presence of the synthase IIa (SSIIa) allele proves advantageous in augmenting the amylose content, thereby resulting in heightened firmness and resistant starch content in pasta [38]. However, the performance of synthase IIa (SSIIa) is reliant on available resources. Developing wheat varieties suited for noodles and pasta production remains a multi-dimensional and time-intensive endeavor. The impact of genes on plant performance is notably intricate. For example, the Wx-B1 allele not only influences amylose levels but also impacts granule size. Moreover, the structure and functional attributes of starch are also influenced by environmental variables, such as heat and drought stress [105].

### 6.2. Adding Exogenous Starch

The exogenous starch is extensively employed to modulate the quality of noodles and pasta. In generally, native starch (corn starch, potato starch, cassava starch, high-amylose corn starch, etc.), physically-modified starch (pre-gelatinized starch, retrograded starch), and chemically-modified starch (cross-linked starch, esterified starch, etc.) are used as additives in the preparation of noodles and pasta, commonly incorporated at levels below 40% relative to the weight of wheat flour (Table 3). The effect of these exogenous starches on color, cooking quality, texture property, and even on the carbohydrate digestibility of the resulting noodles and pasta has been investigated previously [106,107,108,109]. Research centered on the influence of exogenous starch on noodles and pasta quality are summarized in Table 3.

The brightness of noodles and pasta is enhanced subsequent to the incorporation of exogenous starch [114,115,116], with the exception of banana starch and acetylated potato. The a* and b* values of noodles commonly exhibit a decline or minimal variations following the introduction of exogenous starch, except for diphosphated cross-linked high amylose starch [114,115,116,117]. These variations are associated with the dilutive influence of exogenous starch [115]. The addition of banana starch leads to a reduction in the brightness of the pasta, indicated by decreased L* and ΔL* [108]. Meanwhile, the L* of the instant noodles displays decreasing trends upon the incorporation of acetylated potato starch in contrast to the control sample [115]. The cooking qualities of noodles and pasta are influenced by the introduction of exogenous starch. In general, the cooking time of noodles and pasta tends to be reduced with the addition of exogenous starch [107,110]. The shortened cooking time can be attributed to the diluted gluten content following the introduction of exogenous starch into the noodles [114]. This dilution of gluten consequently leads to a diminished barrier effect surrounding the starch [95]. Conversely, incorporating starches with elevated levels of amylose, such as cross-linked high-amylose corn starch and retrograded starch, would prolong the cooking time of noodles due to the heightened amylose content [106]. In contrast to the influence on cooking time, the introduction of exogenous starch results in an increase in the cooking loss of noodles and pasta [106,108,110,111]. This phenomenon can be ascribed to the diminished integrity of the gluten network architecture within noodles and pasta subsequent to the incorporation of exogenous starch [108,114]. Nevertheless, the cooking loss of white salted noodles diminishes as the amount of acylated potato or sweet potato starch reached 20%. This contradictory effect of acylated starch on cooking loss of noodles is associated with the derivatization process on starch [120].

The textural properties of noodles and pasta are additionally influenced by the incorporation of exogenous starch. The firmness of noodles and pasta exhibits a diminishing trend with the present of exogenous starch [107,108,110,114]. The softened texture of noodle (pasta) may be ascribed to the decreased gluten content and enhanced starch swelling capability [44]. Moreover, the introduction of physically altered starch (gelatinized-retrograded and extruded starches) could elevate the stickiness of noodles or pasta. Conversely, the incorporation of acylated potato starch and acylated sweet potato starch proves advantageous in enhancing the elasticity, extensibility, and the slipperiness of instant noodles and wheat noodles [115,120]. This favorable impact of acylated starch on the texture characteristics of noodles may be attributed to acylation’s effect on the interactions between starch chains by means of steric hindrance. Steric hindrance could potentially modify the starch’s hydrophilicity, hydrogen bonding, and swelling behavior [120]. The sensory characteristics of salted noodles exhibits a rising trend following the introduction of external starches, such as potato starch, corn starch, and pre-gelatinized starch [110,112]. This increase could be ascribed to the beneficial influence of exogenous starch on noodles color, softness, and smooth texture [112]. Moreover, the integration of exogenous starches results in a decreased sensory score of the noodles, particularly at higher concentrations [82,110,111]. The inclusion of exogenous starches, likewise, diminishes the sensory assessment of pasta due to decreased firmness [82], while the incorporation of banana starch enhances the sensory quality of the pasta [108]. The addition of endogenous starch (banana, high-amylose maize; cross-linked high-amylose maize) can elevate the resistant starch content of noodles and pasta, thereby enhancing the nutritional value of the end product [106].

Typically, the addition of exogenous starch tends to dilute gluten content, resulting in heightened brightness, increased cooking loss, and reduced firmness. On the contrary, incorporating acetylated potato starch enhances the hardness and the elasticity of instant noodles. Exogenous starches indeed exert a noticeable impact on the quality of noodles and pasta. Further research is warranted to select starch properties that enhance the quality of noodles and pasta.

### 6.3. Others

To improve the quality of noodles, diverse techniques can be employed to refine starch characteristics during the noodle-making procedure. Both physical and biological approaches have been utilized to alter starch properties in noodles, among which high temperatures stand out as a prevalent modification method. For example, steaming can induce a partial gelatinization of starch in noodles, elevating both the cooking quality and the texture of the product [121]. The high-temperature dry process is extensively employed in the production of pasta and noodles. This method notably reduces cooking losses while enhancing the firmness of noodles and pasta through its impact on starch properties [78,81,83]. Simultaneously, high-temperature treatments foster the cross-link of protein, which has important effect on quality of noodles.

In recent years, biological methods such as enzyme and fermentation have been used to modify starch structural and functional properties for quality regulation in noodles and pasta. For example, the appropriate addition of α-amylase can enhance the cooking and textural qualities of noodles and pasta by moderately breaking down starch molecules [122,123,124]. Fermentation emerges as another effective technique for enhancing noodle quality, given its impact on starch structure, functional properties, and starch–protein interactions [125]. Our prior research noted that alterations in starch properties, induced by fermentation during processing, exert a profound influence on the quality of Chinese hand-stretched dried noodles [79]. Consequently, fermentation represents a promising method for improving the quality of wheat-based noodles.

## 7. Conclusions

The impact of starch on the quality of noodles and pasta is noteworthy. Extensive examinations have been conducted on how starch from varying sources influences the quality of noodles and pasta, while the variations in the structural and functional properties of starch also have considerable impact on starch–gluten interactions (physical and chemical interaction), thereby altering the quality of noodles and pasta. Comprehending the intricate mechanism underpinning the quality of noodles and pasta proves challenging when solely concentrating on starch.

Additionally, the characteristics of starch undergo changes during the noodle manufacturing process, particularly with the adoption of modern processing techniques such as high-speed dough mixing, resting, fermentation, drying, and storage. Nevertheless, the adjustments in starch properties during noodles and pasta production and their subsequent effects on noodles have been largely disregarded. Understanding the impact mechanism of starch property changes triggered by various processing stages is vital for effectively overseeing and elevating noodles and pasta quality throughout production.

## Figures and Tables

**Table 1:** Effect of the amylose content on the quality of wheat flour-based noodles and pasta.

Type of Noodles	AM Content (%)	Changes on the Quality of Noodles/Pasta with Increased AM Content	Reference
Noodles	-	Negative for sensory quality	[8]
White salted noodles	27–32	Increased hardness	[31]
White salted noodles	3.0–26.5	Increased cooking time and hardness Decreased water uptake, springiness, and cohesiveness	[36]
Pasta	28.2–54.3	Decreased cooking times, water absorption, and springiness Increased firmness, gumminess, chewiness, cooking loss, and resistance to overcooking	[37]
Pasta	22–35	Increased firmness (SSIIa-Ab null flour), cooking loss, and resistance to overcooking	[38]
Pasta	-	Increased cooking quality and resistant starch	[39]
Noodles	17.5–28.4	Increased cooking time, decreased springiness	[40]
Dry noodles	0.6–24.1	Positive for color, appearance, stickiness, and total sensory score	[41]
Noodles	36.7–93.3	Increased hardness, decreased springiness and cohesiveness	[42]
Pasta	33.3–57.8	Decreased water absorption and glycemic index Increased cooking loss	[43]
White salted noodles		Increased hardness and cohesiveness	[44]

-, signifies that the data that were not reported. AM: Amylose.

**Table 3:** Effect of exogenous starch on the quality of wheat flour-based noodles and pasta.

Type of Starch	Adding Amount (% Based on Wheat Flour)	Type of Noodle	Influence	Reference
Native starch				
Banana starch	5–20	Pasta	Darkened the color of pasta Increased cooking loss and sensory quality of pasta Decreased the firmness of pasta	[108]
Corn starch	5–15	White salted noodle	Increased cooking loss, cooking weight, and the sensory quality of noodles	[110]
Potato starch	10–40	Noodles	Increased cooking loss and adhesiveness, springiness, and broken ratio Decreased sensory score	[111]
Potato starch	0–30	Fresh noodles	Increased water absorption, cooking loss, breaking rate, hardness, adhesiveness, sensory score (slight increase) Decreased springiness, and cohesiveness	[112]
High amylose maize starch	0–35	pasta	Reduced the in vitro starch digestibility	[106]
Sweet potato starch	10–30	Pasta	Decreased cooking time, firmness, resistant content, a* and sensory score Increased stickiness and L*	[82]
Physical modified starch				
Gelatinized-retrograded starch	10–20	noodles	Increased cooking loss Reduced water uptake, hardness, adhesiveness, and gumminess	[107]
extruded starches				
Pre-gelatinized corn starch	5–15	Salted noodles	Increased cooking loss and the sensory score of noodles Decreased the cooking time of noodles	[110]
Extrusion-cooked corn starch	5–10	Noodles	Increased the tensile of dough Increased cooking loss and smoothness	[113]
Chemical modified starch				
Hydroxy propylated Tapioca	10–30	Noodles	Lowered Young’s modulus and the breakability of the noodle	[109]
Cross-linked waxy maize starch	10–30	Non-fried instant noodles	Decreased rehydration time, hardness, and a* Increased L*, cooking loss, and water absorption	[114]
Acetylated potato starch	10–20	instant fried noodles	Increased L*, the hardness of noodles Decreased a* and b*	[115]
Esterified wheat starch	4	White noodles	Increased the L, b*, water absorption rate, adhesiveness, and stretching distance Decreased a*, hardness cooking loss, springiness,	[116]
phosphorylated cross-linked wheat starch	0–10%	Pasta	Decreased water uptake, firmness, glycemic index and L* Increased cooking loss	[117]
Acid-thinning corn starch	5–15	Salted noodles	Increased the cooking loss of noodles Decreased cooking time, cooking weight, and the sensory score of noodles	[110]
Esterified tapioca starch	6	Japanese white salted noodles	Enhanced softening Decreased cooking time	[113]
Cross-linked phosphorylated RS4 wheat starch	10–40	white salted noodles	Decreased extensibility, cohesiveness, and the springiness of noodles	[118]
Diphosphated cross-linked high amylose starch	-	Pasta	Increased a*, cooking time, resistant starch content, Decreased L*	[119]

-, signifies that the data were not reported.
